# mitoLEAF: mitochondrial DNA Lineage, Evolution, Annotation Framework

**DOI:** 10.1093/nargab/lqaf079

**Published:** 2025-06-11

**Authors:** Nicole Huber, Noah Hurmer, Arne Dür, Walther Parson

**Affiliations:** Institute of Legal Medicine, Medical University of Innsbruck, Innsbruck 6020, Austria; Institute of Legal Medicine, Medical University of Innsbruck, Innsbruck 6020, Austria; Institute of Mathematics, University of Innsbruck, Innsbruck 6020, Austria; Institute of Legal Medicine, Medical University of Innsbruck, Innsbruck 6020, Austria; Forensic Science Program, The Pennsylvania State University, University Park, PA 16802, United States

## Abstract

The study of mitochondrial DNA (mtDNA) provides invaluable insights into genetic variation, human evolution, and disease mechanisms. However, maintaining a consistent and reliable classification system requires continuous updates. Since Phylotree updates ended in 2016, the accumulation of new haplogroup findings in individual studies has highlighted the critical need for a centralized resource to ensure consistent classifications. To address this gap, we present mitoLEAF, a collaborative, freely accessible, and academically driven repository for mitochondrial phylogenetic analyses. Unlike commercial alternatives that restrict access to their customers through subscription or purchase, mitoLEAF is openly accessible and replicable, ensuring transparency and scientific reproducibility. Hosted as a GitHub repository and supported by an interactive website, mitoLEAF provides an evolving, quality-controlled phylogenetic resource derived from GenBank, EMPOP, and peer-reviewed literature. In this first release, it expands the haplogroup landscape from 5435 to 6409 haplogroups, integrating recent findings and improving phylogenetic accuracy. By excluding known pathogenic variants, mitoLEAF aims to mitigate ethical concerns associated with reporting medically relevant variants. By prioritizing open science over commercial interests, mitoLEAF serves as a vital, community-driven platform for mitochondrial research, fostering collaboration and continuous development.

## Graphical Abstract

**Graphical Abstract:**
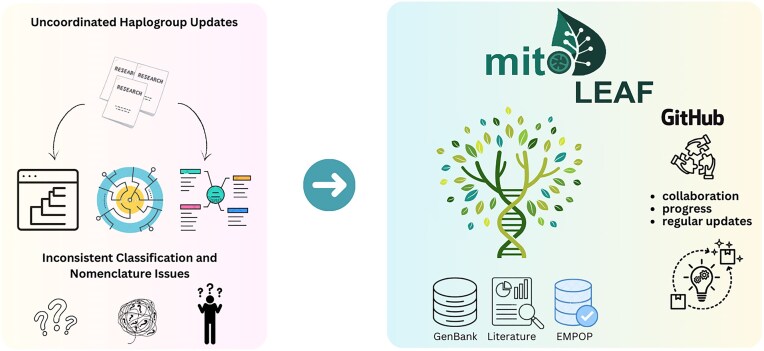

## Introduction

Mitochondrial DNA (mtDNA) has emerged as a valuable marker for investigating human evolutionary history, population genetics, and medical genetics. Due to its unique characteristics, including a high copy number, maternal inheritance, and a rapid mutation rate [1–3], mtDNA provides valuable insights into the deep branches of our genetic ancestry. The field of mtDNA phylogenetics focuses on deciphering the complex relationships between individuals and populations based on the analysis of haplogroups [4, 5].

Haplogroups represent distinct branches within the mtDNA phylogenetic tree, capturing the genetic diversity and ancestral lineages of populations across different geographic regions [6]. The development of Phylotree [7] significantly advanced the classification and delineation of haplogroups, serving as a fundamental reference for the mitochondrial genetics community. However, there is no universal rule for defining when a branch should be formally recognized as a haplogroup, and different studies apply varying criteria. The absence of a standardized definition has led to inconsistencies, particularly since Phylotree ceased updates in 2016, resulting in independently proposed haplogroups and fragmented nomenclature. This lack of standardization hinders comparative studies and underscores the need for reliable tools to ensure accuracy and consistency in haplogroup estimation. Until such a consensus is established, mitoLEAF maintains compatibility with existing classifications [8, 9] while integrating recent refinements.

While some commercial services, such as FamilyTreeDNA (https://www.familytreedna.com/), have developed their own mtDNA trees, these proprietary resources are only accessible to paying users, limiting their utility for open scientific research. In contrast, mitoLEAF was developed as a freely accessible, community-driven alternative that ensures transparency, replicability, and ongoing academic contributions. By offering unrestricted access, mitoLEAF allows researchers across disciplines to engage with a high-quality, continuously updated phylogeny without financial or institutional barriers.

To ensure accurate haplogroup classification and phylogenetic placement within mitoLEAF, robust analytical tools are essential. One such widely recognized tool is SAM2 [10], initially developed for forensic genetics but applicable across multiple disciplines. SAM2 is implemented in the European DNA Profiling Group’s Mitochondrial DNA Population Database (EMPOP) [11] and is recommended by the International Society for Forensic Genetics (ISFG) [12]. By providing a standardized framework for haplogroup estimation, SAM2 ensures consistent classification across diverse datasets, making it a critical resource for forensic genetics, evolutionary biology, and medical research. Despite these analytical advancements, the volume of newly sequenced mtDNA genomes (mitogenomes) continues to exceed the capacity of efforts to standardize and integrate haplogroup classifications.

### Challenges in mtDNA phylogeny research

**Figure 1.**
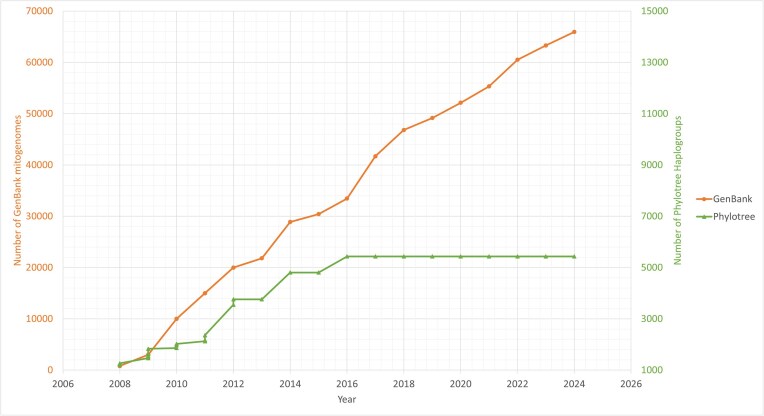
Growth of mitogenomes in GenBank compared to growth of Phylotree haplogroups.

As new mitogenomes continue to accumulate in the NCBI GenBank database, they provide valuable data for refining the human mtDNA phylogeny. Figure 1 illustrates the exponential growth of mitogenomes in GenBank compared to the stagnation in Phylotree updates, emphasizing the need for continuous updates to both the dataset and the phylogeny.

Despite the extensive availability of mtDNA data, the lack of a centralized framework has led to a natural diversification of haplogroup definitions through independent research efforts [13–21]. While these studies have contributed valuable insights, the resulting decentralized structure has made it difficult to maintain a unified phylogeny. This uncoordinated expansion has presented several challenges currently affecting the research community.

*Decentralized publication*: Many studies proposing new haplogroups are published in supplementary materials, making them difficult to access and integrate into a unified phylogeny. There is no common format to publish new haplogroups although it would make sense to publish the entire tree in a common format (e.g. Newick, nexus, phylip) that allow easy integration or downstream analysis. As a result, most published updates are not widely incorporated into broader phylogenetic frameworks due to the lack of a standardized integration process and the additional effort required for manual curation.

*Limited datasets*: Some studies rely on limited datasets, potentially compromising the accuracy and generalizability of their findings. For example, Maier *et al.* [14] used data that were deemed questionable in another study [22], which is why these sequences are usually not used for phylogenetic tree construction. This caution is necessary, as integrating external data requires rigorous quality assessment to prevent the incorporation of potentially unreliable sequences.

*Inconsistent methodologies*: Variations in analytical approaches and criteria for defining haplogroups contribute to inconsistencies and discrepancies. This makes it difficult to simply combine all updates from literature.

*Insufficient quality control*: Varying quality control measures can introduce errors and biases into haplogroup identification. Groups with limited prior experience in this specific area define new haplogroups that are questioned later. The findings in study [16] illustrate this issue, as they were subsequently challenged by a study [23] claiming artificial recombination. This dispute has resulted in conflicting definitions of haplogroup B2b9d in the current literature.

*Conflicting nomenclature*: Nomenclature conflicts further complicate reliable haplogroup identification. Researchers often face conflicting definitions for the same haplogroup, making it difficult to choose the appropriate resource. For example, haplogroup T2e3 is defined by mutation T13962C in the YFULL Mtree database (https://www.yfull.com/mtree/T2e/) but by mutation 9947A in [24]. This inconsistency forces researchers to choose a specific nomenclature system, hindering consensus, particularly given the prevalence of similar discrepancies.

Another challenge in haplogroup definition arises from ambiguities in phylogenetic alignment, particularly the distinction between 5′ and 3′ alignment approaches. A previous study [13] proposed a subclade of N1a1a3, defining it by the variants A5747d and T11087C, using 5′ alignment. However, analysis using SAM2, which applies a 3′ phylogenetic alignment [25, 26], indicated that the 3′ phylogenetic representation of the deletion is 5752del rather than 5747del. This discrepancy highlights a broader challenge in mitochondrial phylogenetics: the use of alternative left- or right-aligned indels, leading to variations in variant annotation. Such variations can influence haplogroup definition and subsequent phylogenetic analyses. If a different deletion site is used to define a subclade, it can lead to discrepancies in phylogenetic placement, potentially resulting in subclades that do not fully reflect established evolutionary relationships. These variations can propagate through databases and subsequent research, complicating the harmonization of classifications across literature. Notably, Mitomaster [27] has implemented the 3′ notation following the recommendations of the Human Genome Variation Society, the internationally recognized standard for the description of DNA, RNA, and protein sequence variants [28].

To address the challenges posed by the discontinuation of Phylotree and to build upon the advancements introduced by tools like SAM2, we present mitoLEAF. Built upon publicly available data, mitoLEAF offers a comprehensive and up-to-date human mitochondrial phylogenetic tree, free of known pathogenic variants. Furthermore, it provides valuable metadata, such as publication information, data on geographic origin, and details regarding sequencing technology and assembly. The user-friendly interface and standardized data format facilitate exploration and individual downstream analyses, addressing the limitations of previous resources.

## Materials and methods

### Phylogeny update and data integration

A systematic literature review identified newly defined mtDNA haplogroups. The NCBI PubMed database was searched using the keywords “mtDNA,” “haplogroups,” “phylogeny,” and “species: human,” with results restricted to publications from 2016 onward. Titles and abstracts were manually screened for relevance, and haplogroups were extracted from supplementary data or the main text. Extracted haplogroups were then converted to EMPOP format for quality control and downstream analysis.

### Integration of forensic update to phylotree nomenclature

We integrated the published subclades in study [29] by resolving the asterisk (*) nomenclature used to denote changes, adhering to Phylotree’s naming convention. We identified the ancestral haplogroup for each subclade, systematically checking existing subhaplogroups to determine the next available identifier. For instance, W7*, a subclade of W7 with no existing subclades, was designated as W7a. In contrast, T2h*, a subclade of T2h, was assigned T2h3 as T2h1 and T2h2 were already present. The complete list of these reassignments is provided in Supplementary Table S1.

### Retrieval and processing of mitogenome sequences

Mitogenome sequences were obtained from the NCBI Nucleotide database using the search term:

(“Homo sapiens”[Organism] AND 00000016500[SLEN]: 00 000 016 619[SLEN] AND mitochondrion[filter]) NOT (“Homo sapiens neanderthalensis”).

To avoid conflicts with the extended IUPAC nucleotide code, all “D” characters in the retrieved FASTA sequences were replaced with “-”. While “D” in the IUPAC code technically represents A, G, or T, we have found that submitters often use “D” to denote a deletion. Therefore, to maintain consistency and accuracy, we treated “D” as a deletion and replaced it with “-”. Positions flanked by deletions due to truncated FASTA strings were set to “N”. Accession numbers and corresponding modifications of affected samples are listed in Supplementary Table S2.

We initially screened the retrieved sequences, excluding 538 samples, as detailed in appendix S1 of the study [22]. We further excluded samples that exhibited the following characteristics:

- More than 30 missing or unknown nucleotides (“N” or “n”).
- A length less than 16 519 base pairs.
- More than 30 ambiguous symbols.
- A cost value greater than 500.
- Affiliation with a subspecies other than *Homo sapiens*, such as “*Homo sapiens altai*”.
- Are a reference sequence.

Accession numbers for all excluded sequences are provided in Supplementary Table S3.

### Metadata retrieval and standardization

Metadata, including geographic origin and sequencing technology, were retrieved via an NCBI API and standardized. Geographic data were corrected for misspellings (e.g. “Finlnad” to “Finland”), and mapped to standardized country designations (e.g. “Eire” to “Ireland”). Non-country descriptors, such as “French” or “East-Africa” were assigned to countries or excluded if nonspecific. By use of the R package countrycode [30] we validated final entries against a standardized list. Sequencing technology data were grouped into predefined categories without additional processing. Metadata for 1000 Genomes Project samples were obtained from the “sequence.index” file on the project’s FTP server [31]. This standardization was applied within the context of this study and does not extend to the current online version of mitoLEAF.

### Expansion of the database

To supplement GenBank data, we added 13 057 vetted genomes from the EMPOP database (restricted access) and 1479 vetted genomes from published literature previously used in Phylotree but not uploaded to GenBank [32]. Table 1 provides a detailed breakdown of the number of mitogenomes along with their respective identifiers.

**Table 1.** Summary of the sources and number of mitogenomes included in the study, along with examples of the identifiers used for each data source

Data source	Number of samples	mitoLEAF identifier
GenBank	61 295	GenBank accession no. e.g. AP010825.1
Vetted full mitogenomes of EMPOP	13 057	EMPOP accession no. e.g. EMP00847
Vetted full mitogenomes literature	1479	1000 Genomes ID, e.g. HG00096

### Quality control and haplogroup estimation

Haplogroup classification was performed using SAM2, a standardized haplogroup estimation tool implemented in EMPOP and recommended by the ISFG. SAM2 provides multiple outputs that aid in haplogroup assignment and phylogenetic placement [9, 26]:

*Phylogenetically aligned haplotype*: A difference-coded haplotype, aligned relative to the revised Cambridge Reference Sequence (rCRS) and structured to reflect known phylogenetic relationships. This output serves as a consistent basis for downstream comparisons and phylogenetic analysis.

*Most recent common ancestor (MRCA) estimation*: The MRCA represents the closest shared ancestor between the queried haplotype and a specific phylogenetic branch [33]. SAM2 identifies this match within the phylogenetic tree, aiding haplogroup assignment.

*Costs*: This value measures how far the observed haplotype deviates from the known phylogeny. Lower costs indicate high-quality data and reliable haplogroup assignments, while higher costs necessitate more careful inspection. Elevated costs may signal sequencing errors or represent a novel, yet undetected branch of the phylogeny.

*Private variants*: Represent differences between the haplogroup motif and the specific haplotype being analyzed. These mutations can be crucial for identifying novel or uncharacterized branches in the phylogeny. However, an unusually high number of private mutations may also indicate potential sequencing errors, data artifacts, or alignment issues.

After sequence alignment using SAM2, we evaluated the overall accuracy of haplogroup assignments by systematically inspecting cost values and private variants.

### Pathogenic filtering

Known pathogenic variants were identified and excluded as part of quality control. The variants, as documented in study [34], were removed from both the 6409 haplogroup motifs and the 61 295 GenBank mitogenomes. Notably, Marshall *et al.* demonstrated that this exclusion does not compromise haplogroup resolution while also ensuring the consideration of private and ethical information.

Additionally, for the GenBank mitogenomes, we retrieved the corresponding PubMed IDs and titles with the aim to determine whether the identified pathogenic variants were primarily associated with studies focused on disease or whether they were also present in studies examining healthy populations.

### Deployment and accessibility

A public GitHub repository (https://github.com/forensicgenomics/mitoLeaf) and a corresponding GitHub Pages site (https://forensicgenomics.github.io/mitoLeaf/) were created to provide access to raw data and allow users to explore the phylogenetic tree and browse haplogroups interactively.

## Results

### Phylogeny update and data integration

A literature review identified several publications proposing new mtDNA haplogroups [13–21, 23, 35], but these were excluded due to data quality concerns and methodological inconsistencies. This study relied on the robust framework established by studies [14, 29], incorporating 943 of the 945 subclades proposed after excluding two samples (F1a1a1*2 and F1a1a1*3) with an excessive number of private variants. All samples from the same unpublished dataset [36] were removed due to insufficient validation (HM436814.1–HM436820.1).

Maier *et al.*[14] introduced the significant L5′7 branch, which we have incorporated into mitoLEAF. However, during our analysis, we observed discrepancies. Maier *et al.* identified “A16183c” as one of the variants within the L5a haplogroup signature. In contrast, our analysis of an internal forensic database and of GenBank mitogenomes revealed that the 16183 deletion (16183del) is more frequent within L5a (sub-)haplotypes, occurring in 55% of cases in the internal database and 61% in GenBank. Consequently, we have revised the L5a haplogroup motif to include 16183del, aligning with our empirical observations. Furthermore, the notation “16183c,” as used by Maier *et al.* to denote an A>C transversion at position 16183, could lead to ambiguity. Within the extended IUPAC nucleotide code, the lowercase “c” can be interpreted as either cytosine (C) or a deletion [37, 38]. To prevent this ambiguity, we reserve lowercase letters solely for expressing extended IUPAC uncertainties.

The L5a2a haplogroup signature, initially defined in [14] by “C309T, G2361A, A15826G,” has been revised and the updated signature retains only 2361A and 15826G. This revision reflects our approach to handling indels in poly-C-stretch regions and the use of phylogenetic alignment. Maier’s alignment “C309T T310C” corresponds to “309del 315.1C,” but since we exclude indels from motif definitions in regions prone to length heteroplasmy [38, 39], the 309del component was removed from the haplogroup signature. Similarly, the L5a2a2a haplogroup signature was updated in the 309–315 region, where the previous notation “T309C!” was removed. All modifications to the haplogroup signatures, in comparison to those published in [14], are summarized in Table 2.

**Table 2.** Modified haplogroup motifs compared to those originally published in study [14]

Haplogroup	Original motif in [14]	Revised motif in mitoLEAF
L5a	455.1T G709A A851G T1822C C5111T G5147A A5656G G6182A T6297C A7424G G8155A A8188G C8582T G9305A G9329A T11025C C11881T G12236A A13105G! A13722G T14212C C14239T T14581C G14905A T14971C G15217A G15884A ***A16183c*** C16355T T16362C	455.1T 709A 851G 1822C 5111T 5147A 5656G 6182A 6297C 7424G 8155A 8188G 8582T 9305A 9329A 11025C 11881T 12236A 13722G 14212C 14239T 14581C 14905A 14971C 15217A 15884A ***16183-*** 16355T 16362C
L5a2a	* **C309T** * G2361A A15826G	2361A 15826G
L5a2a2a	***T309C*!** A464G A1555G C5321T G5460A G5746A G9966A A11182G G16274A A16399G	464G 5321T 5460A 5746A 9966A 11182G C16187 16274A 16399G

Variants are denoted relative to the rCRS.

Moreover, Maier *et al.* excluded positions 152, 195, 310, and 16519. This work, consistent with Phylotree, retained positions 152, 195, and 310.

Integration of the new data resulted in a 17.92% expansion of the mtDNA haplogroup classification system, increasing the total number of haplogroup motifs in mitoLEAF to 6409 from the 5435 present in Phylotree Build 17. This growth incorporates 943 subclades proposed in study [29], and 31 additional haplogroups identified in study [14], enhancing phylogenetic resolution and ensuring a more comprehensive representation of mitochondrial diversity while maintaining compatibility with established nomenclature.

A detailed compilation of the 6409 haplogroup motifs, presented with corresponding haplogroup names and their full signature mutations, is provided in the “Full HG signatures” JSON file. This resource can be downloaded from the downloads section of the project repository, accessible at https://forensicgenomics.github.io/mitoLeaf/downloads.html.

### Sequence retrieval

A total of 63 260 mtDNA sequences were retrieved from GenBank, of which 1965 (3.1%) were excluded based on predefined quality control criteria (Table 3). The final dataset included 61 295 sequences, representing a 152% increase over Phylotree (Supplementary Table S4).

**Table 3.** Exclusion criteria and number of affected samples

Exclusion criteria	Number of samples
From study [22]	538
Questionable and unpublished sequences in study [36]	7
More than 30 “N” or “n”	1396
More than 50 missing bases	6
More than 30 ambiguous symbols	1
Cost values greater than 100	8
*Homo sapiens*sub species	7
Reference sequence	2
Sum	1965

### Metadata retrieval and standardization

**Figure 2.**
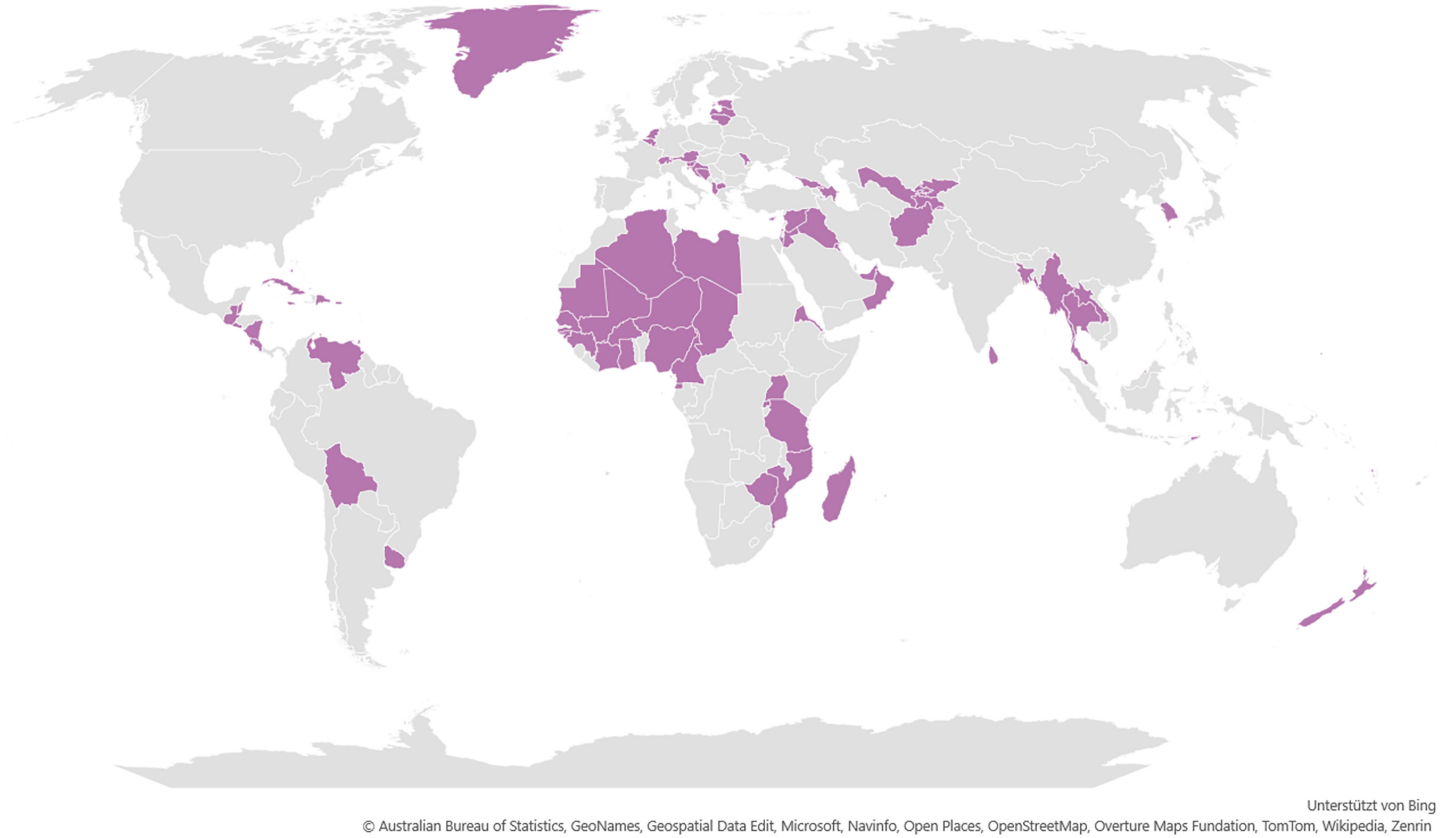
Geographic distribution of countries with ≤50 retrieved mitogenomes. Countries shaded on the map represent underrepresented regions, emphasizing areas with limited mitochondrial genome data. This visualization is based solely on retrieved samples, meaning additional mitogenomes may exist in other databases or without documented geographic origins.

Metadata standardization resulted in geographic origin data for 30 799 sequences, covering 164 countries. While Europe and North America were well represented, coverage remained sparse in parts of Africa, Oceania, and Central Asia (Fig. 2 and Supplementary Table S5). A subset of countries remains completely unrepresented, highlighting gaps in global mitochondrial phylogenetics.

Sequencing technology data were retrieved for 44 356 samples. The primary platforms used were Illumina (*n* = 31 013), Sanger (*n* = 11 556), IonTorrent (*n* = 1508), and Roche 454 (*n* = 227). Samples sequenced using multiple technologies (e.g. “Illumina;IonTorrent”) were grouped and denoted with an asterisk (*). Other technologies, including PacBio and Oxford Nanopore, accounted for 52 samples. No sequencing technology data were available for 16 939 samples. A detailed breakdown of all sequencing technologies is provided within the Supplementary Table S6, while the grouped categories are summarized in Table 4.

**Table 4.** Sequencing technologies retrieved from GenBank

Technology	Count
Illumina	30 499
Illumina*	514
Sanger	11 326
Sanger*	230
IonTorrent	1508
Roche 454	72
Roche 454*	155
Other	52
No data	16 939
Sum	61 295

### Quality control and haplogroup estimation

#### Haplogroup estimation and distribution

Clustering of the 61 295 sequences into the major lineages (L, M, N, and R) resulted in a distribution of 10.6%, 21.2%, 13.4%, and 54.7%, respectively. Within these lineages, we observed extensive diversity, identifying 5899 distinct haplogroups. Among them, the most common were B4a1a1b (596 occurrences), B4a1a1 (469 occurrences), and H3 (453 occurrences). The most frequently observed haplogroups were identified for each major lineage. Table 5 lists the top three haplogroups per lineage along with their frequency and phylogenetic classification (L0–L7, R, M, and N).

**Table 5.** Top 3 haplogroups per lineage

Haplogroup	Count	Lineage	Phylopath
B4a1a1b	596	R	L3-N-R-R+16189-B4
B4a1a1	469	R	L3-N-R-R+16189-B4
H3	453	R	L3-N-R-R0-HV-H-H3
M7c1c3	435	M	L3-M-M7
C1b	277	M	L3-M-M8-C-C1
E1a1a1	228	M	L3-M-M9-E
A2+ (64)	239	N	L3-N-A-A2
I2	109	N	L3-N-N1-I
I4a	99	N	L3-N-N1-I
L2a1b1a	239	L2	L2
L0a2a2a	205	L0	L0
L1c2a1a	55	L1	L1
L4b2a2	15	L4	L4
L5a2a1a	6	L5	L5
L6b	5	L6	L6
L7a	2	L7	L7

This table lists the top three representatives from major lineages R, M, and N, along with representatives from lineages L0 to L7. Counts indicate the frequency of each haplogroup, and Phylopath indicates their phylogenetic placement.

To ensure phylogenetic consistency and facilitate analysis, haplogroups were grouped into broader phylogenetic clusters based on their evolutionary relationships. Two hierarchical clustering approaches were employed:

- *First-level splits*: Haplogroups were grouped based on the first letter and number, such as A1, L0, and H20, resulting in 370 distinct subclades.
- *Macrohaplogroup clustering*: Haplogroups were clustered into broader phylogenetic groups based on their primary letter or major haplogroup designation [A, C, D, E, F, G, H, HV, I, J, K, L0, L1, L2, L3, L4, L5, L6, L7, M, N, O, P, Q, R, R+16189 (often designated as haplogroup B), R0, S, T, U, V, W, X, Y, Z], forming macrohaplogroups that encapsulate multiple sublineages.

All subsequent analyses refer to these defined clusters rather than individual haplogroups, ensuring consistency in phylogenetic interpretation.

**Figure 3.**
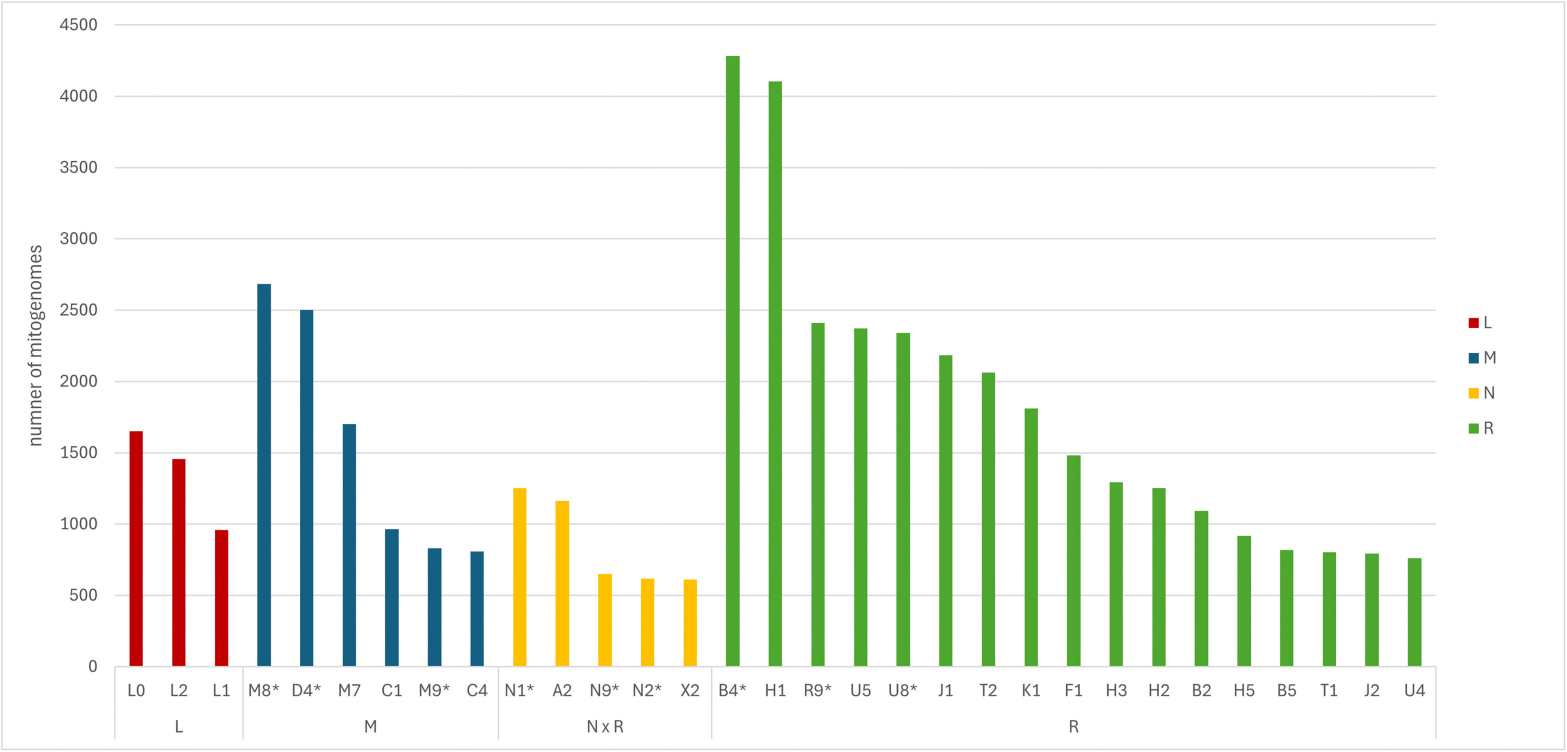
Distribution of first-level splits across lineages (L, M, N, R). Counts for each cluster are displayed on the *y*-axis, and lineages are distinguished by color. A * after a cluster name (e.g. M8*) indicates that this cluster contains additional subclades. For example, M8* includes subclades C and CZ.

Figure 3 summarizes the most frequent (count > 600) first-level splits. For lineage L, the most common splits were L0 (*n* = 1650), L2 (*n* = 1457), and L1 (*n* = 958). Within M, M8* (*n*= 2683) and D4* (*n* = 2501) were most frequent; for N, N1* (*n* = 1252) and A2 (*n*= 1161); and for R, B4* (*n* = 4282), H1 (*n* = 4104), R9* (*n* = 2410), and U5 (*n* = 2372). A complete table of first-level splits is provided in Supplementary Table S7.

### Mapping haplogroup estimations with origins

To visualize haplogroup cluster distributions, we generated world maps for lineages L, M, N, and R, illustrating the geographic distribution of individuals within each cluster and highlighting areas of high and low frequency. Lineage L3, ancestral to lineages M and N, was excluded from this analysis.

Lineage L demonstrated diverse distributions. Cluster L0 was pan-African with Middle Eastern and European presence. L2 showed a broader distribution, including Africa, Europe, and the Americas. L4 was primarily African and Middle Eastern. L5, L6, and L7 were mainly concentrated in East Africa.

Lineage M demonstrated a diverse global distribution. Clusters M and D were prominent, with M showing a broader distribution across Asia, Oceania, and the Americas, while D exhibited a strong presence in East Asia and the Americas.

Lineage N followed a similarly widespread pattern, with notable concentrations in Europe, the Middle East, and North Africa. Clusters J and A were the most prominent, with J being prevalent in Europe, the Middle East, and North Africa, while A extended further into the Americas and East Asia.

Lineage R encompassed the most widely distributed clusters. Cluster H exhibited the highest overall frequency, with a widespread presence across Europe, the Americas, and parts of Asia and Africa. Other clusters within lineage R, such as U and T, also showed a broad geographic distribution.

**Figure 4.**
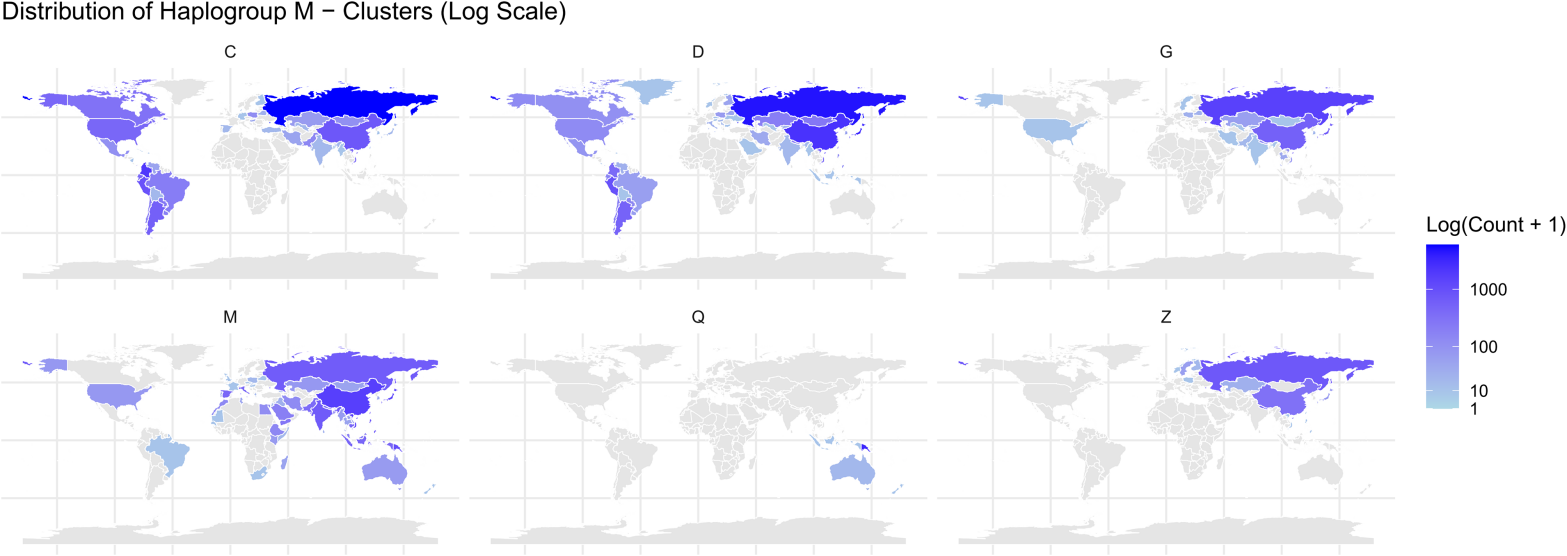
Choropleth map illustrating the global distribution of lineage M, with color intensity representing haplogroup frequency.

The observed patterns are consistent with current literature, reflecting distinct geographic distributions that align with known evolutionary and migratory histories [33, 40–42]. The global distribution of each lineage (L, M, N, and R) is visualized in world maps. Figure 4 presents the map for lineage M as an example, with the complete set available in Supplementary Figs S1–S4.

### Variation in costs across macrohaplogroups

**Figure 5.**
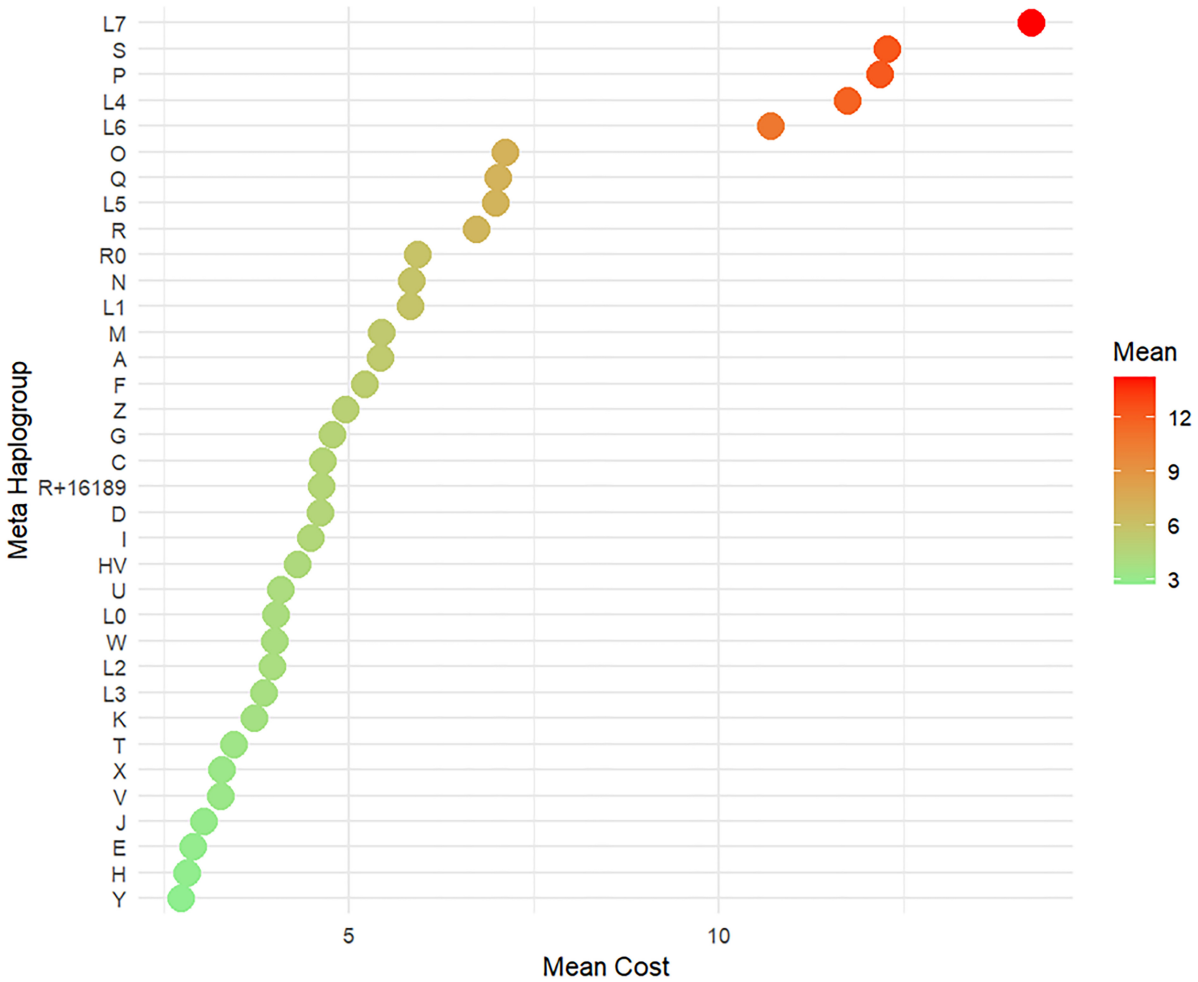
Distribution of mean costs by macrohaplogroup clusters, highlighting elevated costs in L4, L6, L7, P, and S.

Cost values, reflecting sequence deviation from known phylogenies, provide a measure of confidence for haplogroup assignments (Fig. 5). Our analysis revealed a wide range of mean and median cost values across macrohaplogroup clusters, indicating varying levels of divergence or potential sequencing inconsistencies. While the overall dataset had a mean cost of 4.21 and a median cost of 2.99, individual meta-haplogroups ranged from 2.73 (Y) to 14.20 (L7) for mean cost, and from 1.99 (Y) to 13.20 (S) for median cost. The highest costs were observed in L7, P, and S, while the lowest were found in Y, H, and E.

Supplementary Table S8 provides the specific mean and median values for each cluster.

### Deviation from estimated haplogroups

The dataset was categorized into four cost groups based on deviation from the estimated haplogroup: minimal (0–10), moderate (10.01–29.96), substantial (30.03–49.12), and extensive (51.38–93.85) deviation. Most profiles (56 322; 91.85%) were classified into the minimal deviation group, followed by 4744 profiles (7.74%) in the moderate group, 194 profiles (0.32%) in the substantial group, and 35 profiles (0.06%) in the extensive group, as outlined in Table 6.

**Table 6.** Distribution of mitogenome profiles across four cost categories, defined by their deviation from the estimated haplogroup

Deviation level	Count	Range [from, to]
Minimal	56 322	[0, 10]
Moderate	4744	[10.01, 29.96]
Substantial	194	[30.03, 49.12]
Extensive	35	[51.38, 93.85]

The distribution of private variants across cost categories is summarized in Table 7. The mean and median number of private variants increased progressively with higher deviation levels, ranging from 3.01 and 3 in the minimal deviation category to 31.57 and 31 in the extensive deviation category. Standard deviation values for both cost and private variant counts also increased across categories, with cost variability reaching 12.37 in the extensive deviation group and private variant count variability peaking at 6.69. These results indicate a structured trend in the relationship between deviation level and private variant distribution.

**Table 7.** Mean and median number of private variants across cost categories

Deviation level	Mean number of private variants	Median number of private variants	Standard deviation of cost values	Standard deviation of number of private variants
Minimal	3.01	3	2.49	2.06
Moderate	11.01	10	4.26	3.3
Substantial	22.38	23	4.47	4.33
Extensive	31.57	31	12.37	6.69

### Pathogenic filtering

Excluding known pathogenic variants in mitoLEAF prevented potential privacy concerns associated with disease-related variants while ensuring that haplogroup resolution remained unaffected.

Pathogenic variant filtering of the haplogroup motifs (*n* = 6409) revealed five variants in 0.12% of mitoLEAF samples (*n* = 8) across eight different haplogroups. Of these, four variants were homoplasmic, while only one, 14484Y, was heteroplasmic. Table 8 lists these variants along with their affected haplogroups.

**Table 8.** Pathogenic variants found in 6409 mitoLEAF haplogroup motifs

Name	Variants	Affected profiles	Haplogroups	Lineage
Homo3	1555G	2	H24a3, L5a2a2a	R, L5
Homo28	11778A	3	J1d2b, T3, X2p1	R, N
Homo34	14484C	1	Q3b	M
Homo36	14674C	1	M27b1	M
Hetero76	14484Y	1	J1c1d	R

A detailed analysis of the remaining signature mutations within the relevant haplogroups is summarized in Table 9. Haplogroup signatures remained stable following the removal of pathogenic variants, demonstrating no adverse impact on phylogenetic classification.

**Table 9.** Impact of pathogenic variant removal on haplogroup signatures

mitoLEAF motif	Pathogenic variant	Remaining signature variants	Source
H24a3	1555G	2626C 6398T	[9]
L5a2a2a	1555G	464G 5321T 5460A 5746A 9966A 11182G 16274A 16399G	[14]
J1d2b	11778A	16311C 16362C 689C 9123A 14040A 14280G	[9]
T3	11778A	T195C! 1406C 1829G 4225G 11467G 11778A 13956G 13980A 15509G 16325C	[8]
X2p1	11778A	513A 4093G 8645G 9708C 11778A 16291T	[8]
Q3b	14484C	5460A 8454G 9254G A0750G 12684A 16249C 16362C	[8]
M27b1	14674C	64T 199C 236C 1598A 4775G 5788C 16145A 16223C	[8]
J1c1d	14484Y	16213A	[9]

Variants are denoted relative to the rCRS.

The GenBank mitogenomes exhibited a higher prevalence of pathogenic variants as we detected 39 variants in 0.77% of samples (*n* = 471) across 358 haplogroups. Of these, 26 were homoplasmic and 13 heteroplasmic. Table 10 summarizes the identified pathogenic variants and their frequency.

**Table 10.** Pathogenic variants found in the 61 295 GenBank mitogenomes, sorted by occurrence

Name	Type	Pathogenic variant	Positive profiles	Occurrence
Homo28	Homoplasmic	11778A	190	0.31%
Homo3	Homoplasmic	1555G	84	0.14%
Homo34	Homoplasmic	14484C	71	0.12%
Homo6	Homoplasmic	3460A	29	0.05%
Homo14	Homoplasmic	7471.1C	12	0.02%
Homo36	Homoplasmic	14674C	10	0.02%
Homo7	Homoplasmic	3635A	9	0.01%
Homo35	Homoplasmic	14568T	6	0.01%
Homo2	Homoplasmic	1494T	5	0.01%
Homo19	Homoplasmic	8993G	5	0.01%
Hetero6	Heteroplasmic	3243R	5	0.01%
Homo18	Homoplasmic	8851C	4	0.01%
Homo9	Homoplasmic	3700A	3	0.00%
Homo21	Homoplasmic	9176C	3	0.00%
Homo23	Homoplasmic	9185C	3	0.00%
Homo26	Homoplasmic	10197A	3	0.00%
Homo31	Homoplasmic	14459A	3	0.00%
Homo10	Homoplasmic	3733A	2	0.00%
Homo11	Homoplasmic	4171A	2	0.00%
Homo16	Homoplasmic	7511C	2	0.00%
Homo27	Homoplasmic	10663C	2	0.00%
Homo32	Homoplasmic	14482A	2	0.00%
Hetero18	Heteroplasmic	3460R	2	0.00%
Hetero23	Heteroplasmic	4171M	2	0.00%
Hetero61	Heteroplasmic	11778R	2	0.00%
Homo1	Homoplasmic	616C	1	0.00%
Homo13	Homoplasmic	7445G	1	0.00%
Homo22	Homoplasmic	9176G	1	0.00%
Homo24	Homoplasmic	9205- 9206-	1	0.00%
Homo30	Homoplasmic	13094C	1	0.00%
Hetero20	Heteroplasmic	3733R	1	0.00%
Hetero25	Heteroplasmic	4300R	1	0.00%
Hetero31	Heteroplasmic	5650R	1	0.00%
Hetero33	Heteroplasmic	5703R	1	0.00%
Hetero43	Heteroplasmic	8344R	1	0.00%
Hetero55	Heteroplasmic	9185Y	1	0.00%
Hetero69	Heteroplasmic	13042R	1	0.00%
Hetero71	Heteroplasmic	13513R	1	0.00%
Hetero76	Heteroplasmic	14484Y	1	0.00%

**Figure 6.**
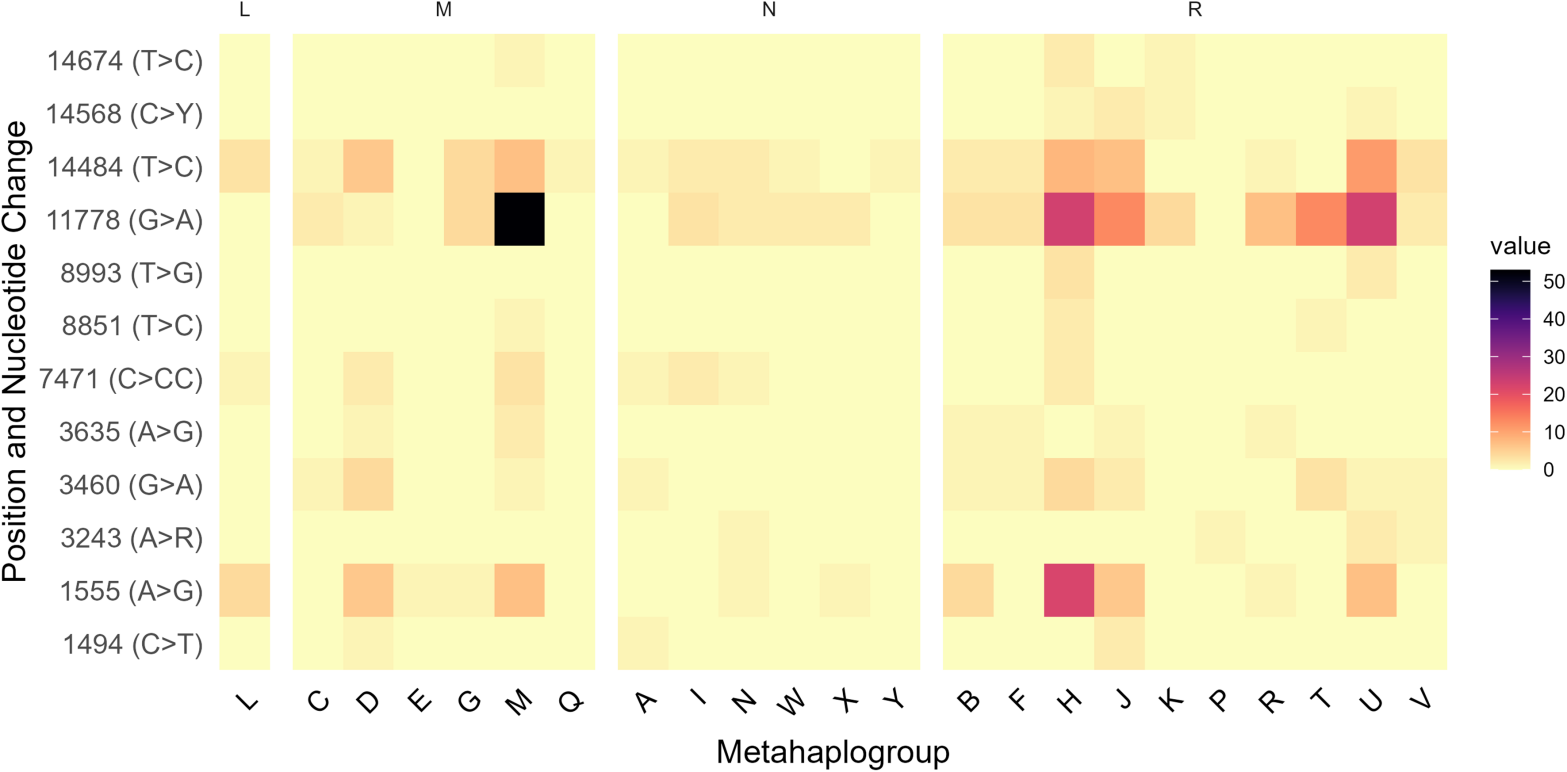
Heatmap of pathogenic variants across major mtDNA haplogroup clusters. The color intensity represents the frequency of pathogenic variants within each haplogroup cluster, with darker shades indicating higher occurrences and lighter shades representing lower frequencies.

We analyzed the more frequent occurring variants with an occurrence threshold ≥0.01% (11778A, 1555G, 14484C, 3460A, 7471.1C, 14674C, 3635A, 14568T, 1494T, 8993G, 3243R, 8851C) regarding their haplogroup distribution. As illustrated in Fig. 6, a heatmap visualization highlights the distribution of these pathogenic variants across major mtDNA haplogroups, revealing patterns of variant accumulation within specific lineages.

### Pathogenic variants in mtDNA haplogroups

Analysis of pathogenic variants revealed 471 sequences containing at least one pathogenic variant. While most samples exhibited a single pathogenic variant, four sequences contained two pathogenic variants, classified as multi-pathogen carrying (mpc) haplotypes. Table 11 lists the mpc haplotypes along with their associated haplogroups and corresponding publication sources.

**Table 11.** Multi-pathogen carrying haplotypes

GenBank ID	Pathogenic variants	Haplogroup	Publication title	Disease-related
FJ015040.1	11778A and 1555G	G2b1a	Co-occurrence of A1555G and G11778A in a Chinese family with high penetrance of Leber’s hereditary optic neuropathy	Yes
JX462687.1	11778A and 1555G	M5	Co-occurrence of m.1555A>G and m.11778G>A mitochondrial DNA mutations in two Indian families with strikingly different clinical penetrance of Leber hereditary optic neuropathy	Yes
JX462739.1	11778A and 1555G	U2e1b	Co-occurrence of m.1555A>G and m.11778G>A mitochondrial DNA mutations in two Indian families with strikingly different clinical penetrance of Leber hereditary optic neuropathy	Yes
MN849780.1	1555G and 14674C	M27b1	Papuan mitochondrial genomes and the settlement of Sahul	No

Notably, 1555G is a ubiquitous variant among the mpc haplotypes, frequently occurring in combination with 11778A. These two variants were also the most prevalent overall (see Table 10). The predominant top-level haplogroups were M (G2b1a, M5, M27b1) and R (U2e1b).

Even though only 39% of publications explicitly focused on disease-related studies, a significant number (60%) of pathogen-carrying haplotypes were identified in broader population genetics research, as detailed in the Supplementary Table S9.

### Deployment and accessibility

mitoLEAF provides a valuable resource for reproducible research and downstream analyses, featuring an intuitive web interface and accessible GitHub repository. Its design incorporates clear color coding (L, red; M, blue; N, yellow; R, green) and interactive elements such as tooltips and clickable, expandable branches, simplifying exploration of the mitochondrial haplogroup hierarchy for both experts and non-experts. The landing page’s circular tree offers a concise overview, with clickable nodes providing access to detailed haplogroup information.

### Explore mitoLEAF: new features for interactive tree exploration

- *Expandable*: Users can expand the entire tree with one click or reset to the default view, making the complex haplogroup hierarchy quickly accessible.
- *Searchable*: The search functionality has been significantly improved compared to Phylotree, making it easier to locate specific haplogroups. For example, searching for “T1” in Phylotree is challenging because “T1” is also a substring of many variants (e.g. T16159C). In Phylotree, searching for “T1” in the JT subtree yields 227 matches, requiring the user to manually scroll through the results to find the desired subtree. mitoLEAF solves this by refining the search to prioritize exact haplogroup matches, greatly reducing effort and improving accuracy.
- *Printable*: The tree can be printed or exported for offline use, making it accessible for presentations, publications, or further offline analysis.

### Browse haplogroups: streamlined access to lineages and metadata

**Figure 7.**
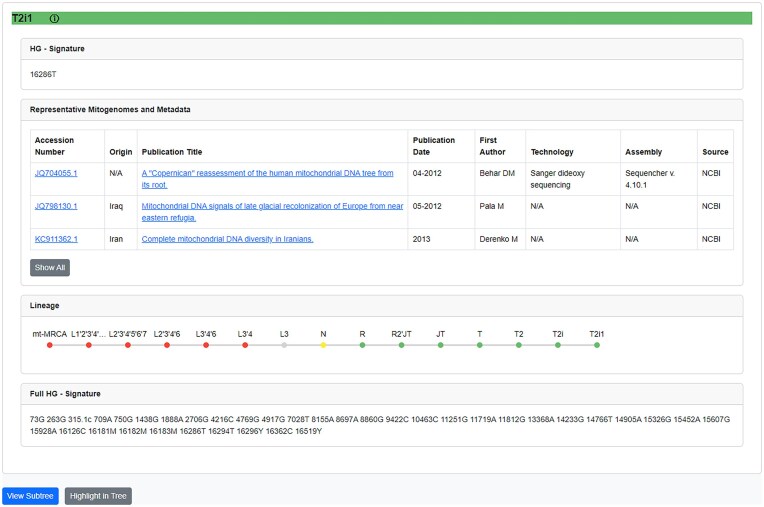
Haplogroup info view of the mitoLEAF website.

The “Haplogroups” page provides a dynamic table with search, filter, and download options, listing haplogroups alongside their defining mutations. Expanding a haplogroup entry redirects users to the “Browse Haplogroups” page (Fig. 7), which provides detailed metadata to facilitate efficient lineage exploration, streamlining access to information traditionally dispersed across multiple platforms. This extended haplogroup information view summarizes lineage metadata, defining mutations, and related sequencing details.

- *HG-Signature*: Displays the branch-defining variant(s), making it easy to identify the haplogroups defining mutation(s).
- *Integrated metadata*: Displays accession numbers, geographic origin, publication details, sequencing technology, and assembly methods. It integrates metadata from major databases such as GenBank, EMPOP, and 1000 Genomes.
- *Complete lineage and descendants*: Visualizes the haplogroup lineage from mt-MRCA to the current node and lists direct descendants for context.
- *Full-HG-Signature*: outlines all variants defining the haplogroup relative to rCRS.
- *Interactive actions*: “View Subtree” and “Highlight in Tree” buttons enable precise visualization and navigation within the tree.

These enhancements represent a significant improvement over traditional tools like Phylotree, consolidating critical information into a single interface and improving accessibility, efficiency, and usability for researchers.

## Discussion

### Phylogeny update and data integration

To ensure phylogenetic reliability, this study prioritized well-supported mtDNA haplogroups based on data quality, methodological consistency, and alignment with established classifications. We incorporated 943 subclades from [9, 29], and 31 haplogroups from [14], which passed stringent quality control and were consistent with existing phylogenetic structure. Although the literature review identified several potential new haplogroups, many lacked sufficient validation due to limited datasets, heterogeneous methods, or inconsistent reporting. Given the variability in quality and reporting, their integration would have introduced additional layers of complexity and uncertainty.

These challenges underscore a broader issue: the lack of standardized criteria for haplogroup definition continues to hinder consistency across studies. A sustainable solution calls for a dedicated working group to establish evidence-based guidelines, resolve nomenclature discrepancies, and balance resolution with interpretability. Such a consortium would improve reproducibility and support robust phylogenetic comparisons across disciplines.

### Dataset expansion, quality control, and population bias

The reliance on GenBank for mitogenomes enhances accessibility but introduces geographic biases. While metadata standardization helped resolve inconsistencies in geographic origin and sequencing technology, a substantial number of GenBank samples lack documented geographic origin. Additionally, GenBank’s dataset reflects a broader bias in mitochondrial research, with disproportionately more studies focusing on European and US populations. As a result, the overall composition of available data is skewed toward these regions, leaving African, Pacific, and Central Asian populations underrepresented. This highlights that the human mtDNA phylogenetic tree remains incomplete, as sequencing efforts in undersampled populations will be crucial for refining its structure. Future research should prioritize expanding data collection in underrepresented regions rather than adding more samples from already well-characterized populations to ensure a more balanced and comprehensive phylogeny.

### Haplogroup deviation levels and private variants

Analysis of haplogroup deviation levels revealed that most sequences (91.85%) exhibited minimal deviation (cost ≤ 10), reinforcing the reliability of current haplogroup classifications. However, a small subset (0.38%) displayed substantial to extensive deviation (cost > 30). In these cases, the accumulation of private variants may reflect rare mutations or uncharacterized lineages, but could also indicate sequencing artifacts or data quality issues. Haplogroups such as L7, P, and S, which showed elevated deviation scores and private variant counts, may represent underrepresented branches of the phylogeny. While private variants can point to novel haplogroups, they also risk reflecting data quality issues, highlighting the need for cautious interpretation.

### Haplogroup frequency distribution

The distribution of haplogroups across major lineages (L, M, N, R) reflects well-established phylogenetic patterns but also highlights key areas for further research [5]. Lineage R exhibited the highest overall diversity, encompassing well-characterized haplogroups such as H3, B4a1a1b, and J1d2b. The predominance of these haplogroups aligns with previous studies documenting their wide geographic distribution across Eurasia and the Americas [6, 43, 44]. Conversely, lineages L and M exhibited a greater proportion of underrepresented haplogroups, particularly within L5, L6, and M27, indicating gaps in global mitochondrial phylogenetic representation. The low frequencies of certain L haplogroups likely result from sampling biases in publicly available datasets rather than true population frequencies as publicly available datasets may overrepresent certain haplogroups, potentially skewing prevalence estimates [45, 46].

### Revisiting mtDNA haplogroup nomenclature

Mitochondrial haplogroup nomenclature has evolved through historical conventions rather than a strictly phylogenetic framework. Some haplogroups (e.g. E, Q, Z) were assigned distinct letters, while others, like M1, M7, and M8, remained subgroups despite their comparable phylogenetic depth. This has led to inconsistencies that can obscure lineage relationships. A common issue in mtDNA nomenclature is the presence of misleading classifications. For example, M72 is a subclade of M, not M7, and B2 is a subclade of B4. Such inconsistencies can lead to incorrect assumptions about phylogenetic relationships and clustering errors. Recent discussions [47], emphasize the need for a standardized nomenclature system to ensure consistency across studies. To address these challenges, we maintain the conventional letter-based classification for consistency and usability but introduce two-level clustering—macrohaplogroups and first-level splits—to improve organization. While this does not fully resolve nomenclature issues, it provides a clearer structure for comparative analyses, balancing practicality with phylogenetic clarity.

### Pathogenic filtering

Beyond classification challenges, another critical factor affecting phylogenetic accuracy is the presence of pathogenic variants, which can obscure haplogroup relationships by introducing medically significant noise. A key contribution of this study is pathogenic filtering, which enhances phylogenetic accuracy by removing known disease-associated variants. These variants do not reflect neutral evolutionary processes and can distort phylogenetic interpretations. By excluding them, mitoLEAF ensures a more precise phylogeny while preserving haplogroup resolution, as demonstrated by [34].

Filtering pathogenic variants from the classification process also addresses significant ethical and legal concerns in mtDNA research, particularly in applied contexts like forensics. While the raw sequence data containing such variants might be publicly available (e.g. in GenBank), standard phylogenetic resources may incorporate these variants into their classification schemes. For example, PhyloTree uses the known pathogenic variant G11778A (associated with Leber’s hereditary optic neuropathy) as a defining marker for haplogroup T3. Reporting a sample as T3 based on such a scheme implicitly links the sample to this specific disease-associated variant. In contrast, mitoLEAF removes G11778A and similar variants from the defining set used for haplogroup assignment. Therefore, reporting a T3 haplogroup assignment derived from mitoLEAF does not carry this direct implication, even if the variant exists in the underlying sequence. This decoupling is crucial because many forensic guidelines restrict the reporting of disease-associated variants discovered incidentally. By ensuring the haplogroup classification itself is not defined by such markers, mitoLEAF aids compliance with legal frameworks and relieves researchers and practitioners (e.g. forensic scientists reporting in court) from the ethical and logistical burden of managing or interpreting sensitive health-related information tied directly to the reported haplogroup label.

### Sustaining collaboration and future updates

The sustainability of mitoLEAF relies on its open access and collaborative nature. By providing a phylogenetic framework, researchers can contribute new data, suggest haplogroup definitions, and enhance visualization tools. Unlike commercial mtDNA databases, such as Mitotree by FamilyTreeDNA, which operates on a subscription-based model and does not publicly disclose all aspects of its curation process, mitoLEAF fosters transparency and continuous refinement through community collaboration. Built on publicly available sequences and rigorous validation, the framework ensures scientific integrity, long-term accessibility, and broad engagement. To establish long-term consistency in mitochondrial haplogroup classification, we advocate for the formation of a working group in mtDNA phylogenetics. This consortium would define standardized criteria for haplogroup assignment, resolve nomenclature inconsistencies, and guide future updates. While such a global effort requires interdisciplinary collaboration, mitoLEAF provides an infrastructure to facilitate its implementation. The platform enables systematic review of newly proposed haplogroups, refinements, and metadata improvements in alignment with expert-driven guidelines. As the governance model evolves, a hybrid approach—integrating expert oversight with community contributions—will ensure that updates reflect both scientific consensus and ongoing advancements in the field.

By bridging the gap left by Phylotree, mitoLEAF provides a curated, community-driven resource that ensures reliable haplogroup classifications while remaining adaptable to future discoveries. Prioritizing academic accessibility over commercial interests, we provide a sustainable and scientifically rigorous alternative to proprietary mtDNA phylogenies. Through collaboration and open access development, mitoLEAF will continue to evolve as a high-quality tool supporting forensic, medical, and evolutionary genetics.

## Supplementary Material

lqaf079_Supplemental_Files

## Data Availability

The code used to generate the GitHub Pages website is available at https://github.com/forensicgenomics/mitoLeaf and is archived on Zenodo: https://doi.org/10.5281/zenodo.15088974. All data required to support the analyses presented in this study are provided as supplementary material. Due to file size limitations, Supplementary Table S4 is archived separately on Zenodo and can be accessed at https://doi.org/10.5281/zenodo.15124031.
