# Supplementary material for: mitoLEAF: mitochondrial DNA Lineage, Evolution, Annotation Framework
Source: NAR Genom Bioinform. 2025 Jun 11;7(2):lqaf079. doi: 10.1093/nargab/lqaf079 (PMC12153335; doi:10.1093/nargab/lqaf079)
Supplement: lqaf079_Supplemental_Files [file lqaf079_supplemental_files.zip › Supplementary_Figures_S1-S4.pdf]

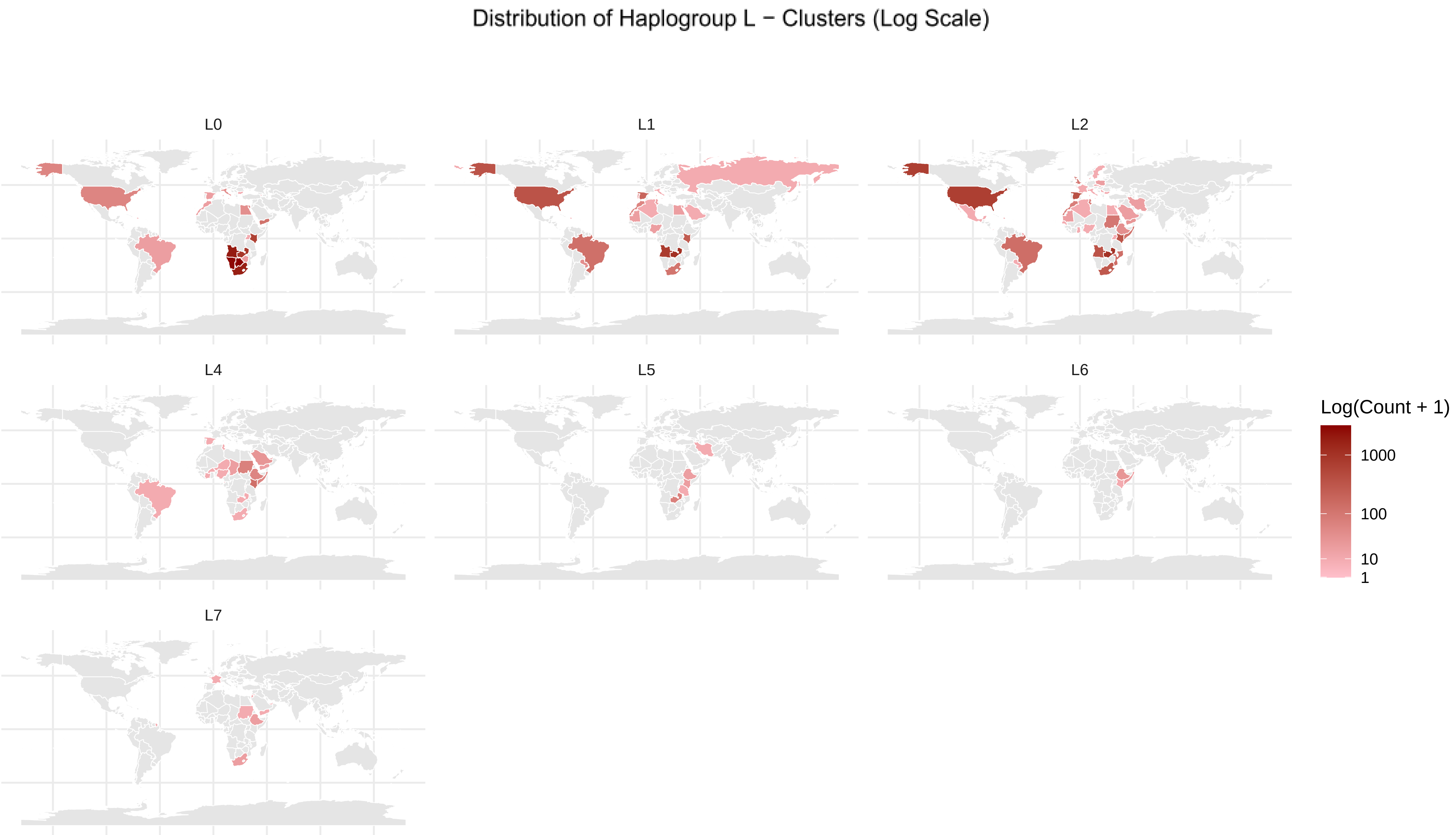

Supplementary Figure S1. Global Distribution of Haplogroup L Clusters (Log Scale).
World map illustrating the geographic distribution of haplogroup L subclades, with counts represented on a logarithmic scale. Darker colors indicate higher frequencies, highlighting regions with greater representation of specific subclades.

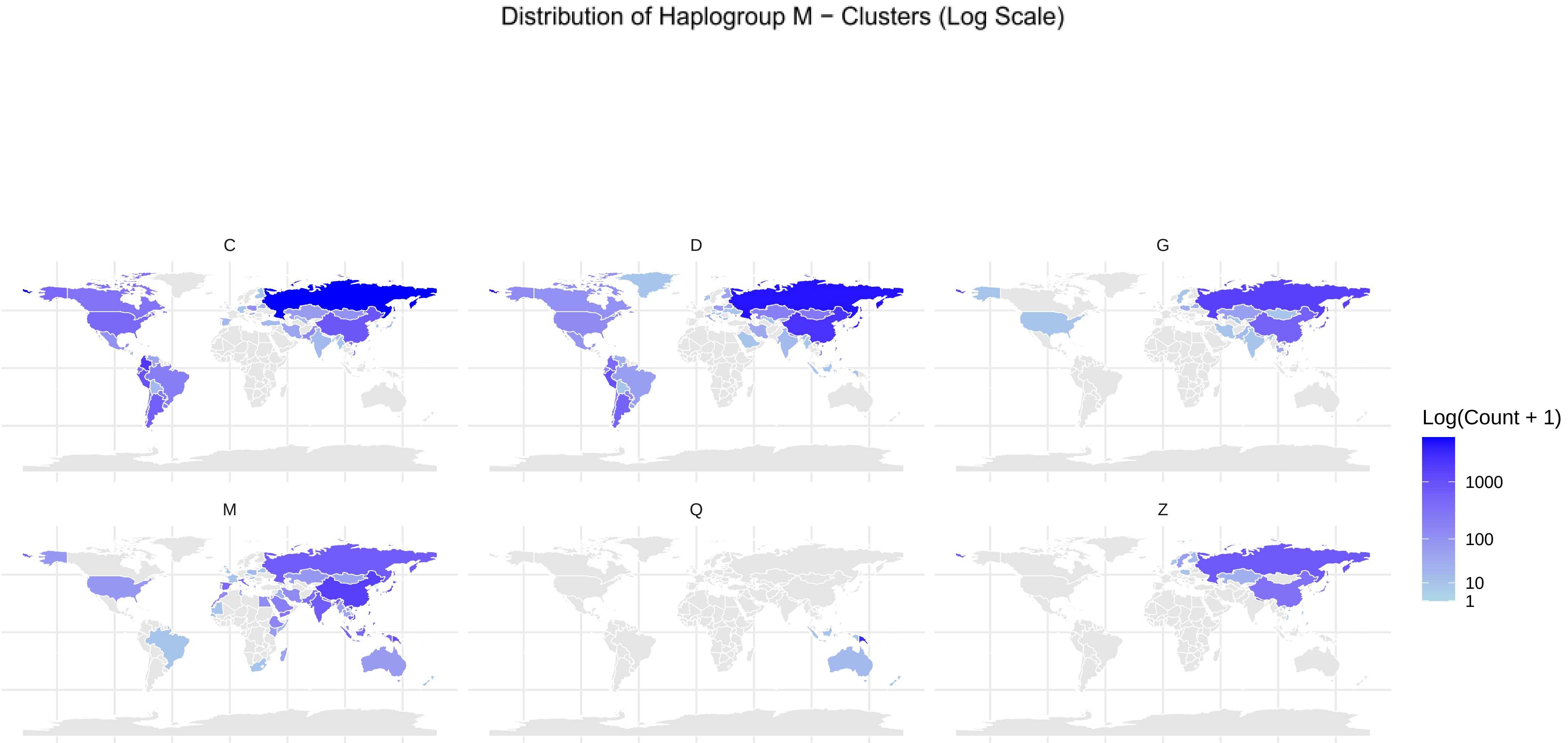

Supplementary Figure S2. Global Distribution of Haplogroup M Clusters (Log Scale).
World map illustrating the geographic distribution of haplogroup M subclades, with counts represented on a logarithmic scale. Darker colors indicate higher frequencies, highlighting regions with greater representation of specific subclades.

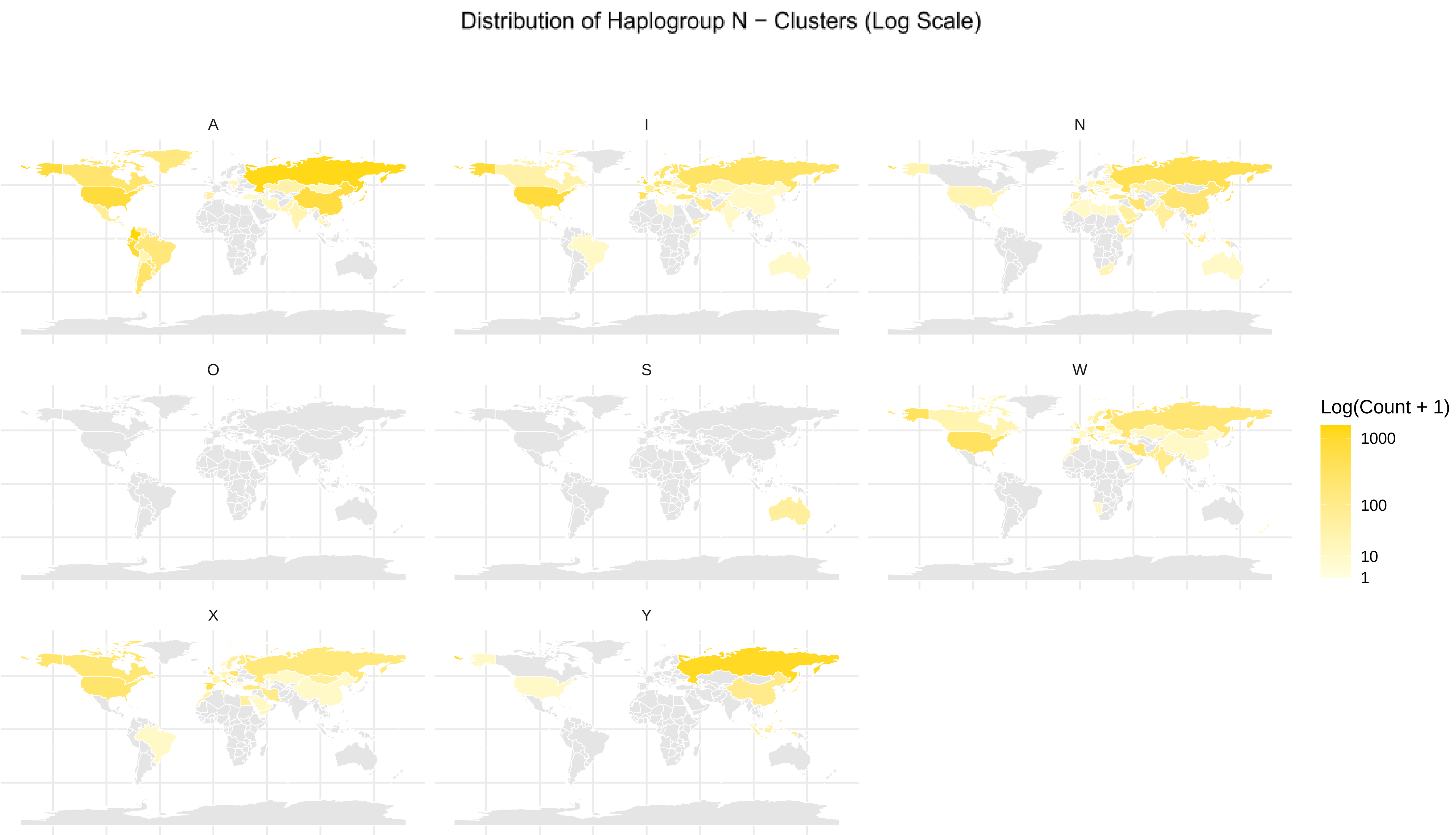

Supplementary Figure S3. Global Distribution of Haplogroup N Clusters (Log Scale).
World map illustrating the geographic distribution of haplogroup N subclades, with counts represented on a logarithmic scale. Darker colors indicate higher frequencies, highlighting regions with greater representation of specific subclades.

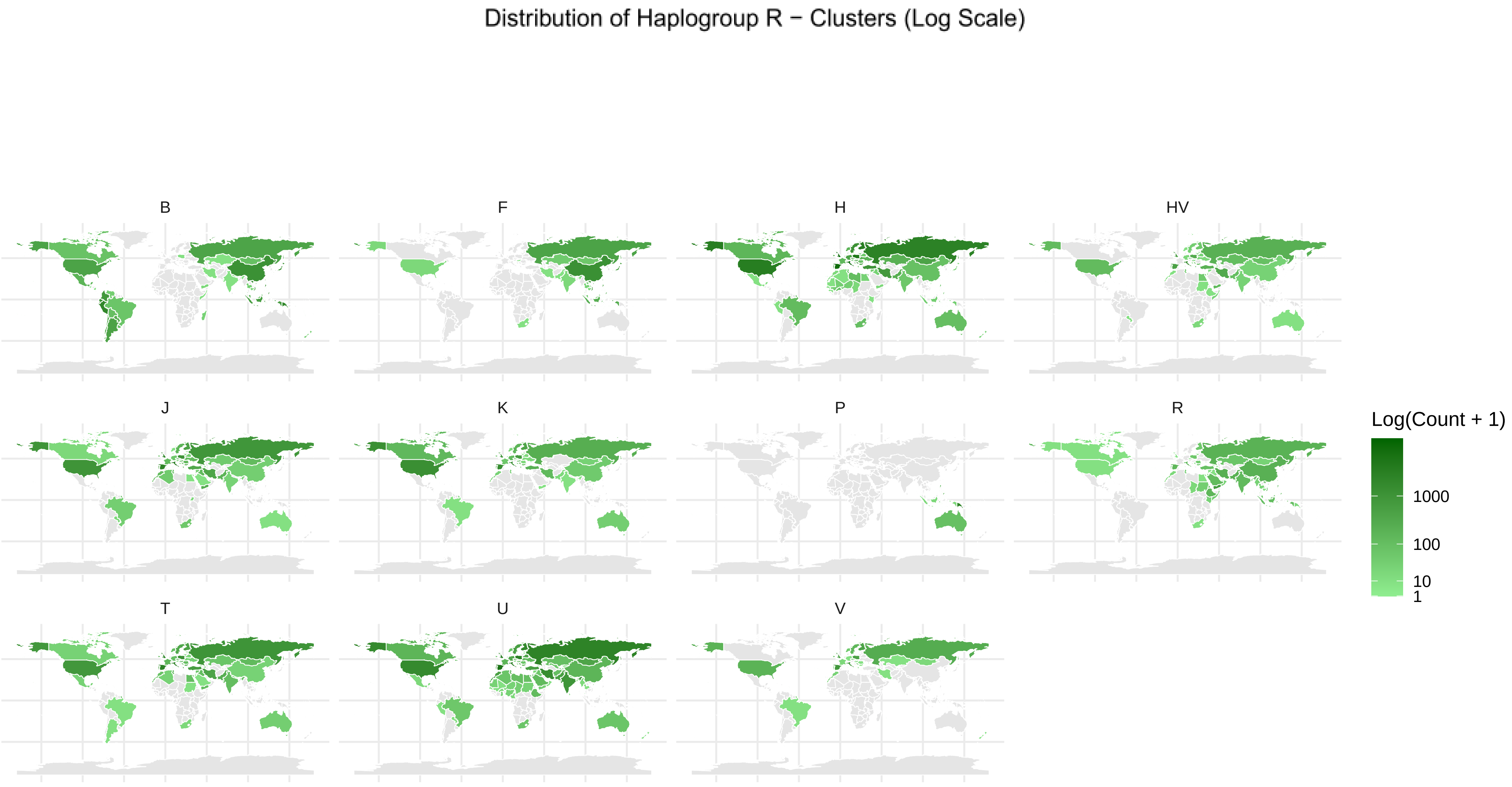

Supplementary Figure S4. Global Distribution of Haplogroup R Clusters (Log Scale).
World map illustrating the geographic distribution of haplogroup R subclades, with counts represented on a logarithmic scale. Darker colors indicate higher frequencies, highlighting regions with greater representation of specific subclades.
